# Altered Expression of Porcine *Piwi* Genes and piRNA during Development

**DOI:** 10.1371/journal.pone.0043816

**Published:** 2012-08-30

**Authors:** Dorota Kowalczykiewicz, Piotr Pawlak, Dorota Lechniak, Jan Wrzesinski

**Affiliations:** 1 Institute of Bioorganic Chemistry, Polish Academy of Sciences, Poznań, Poland; 2 Department of Genetics and Animal Breeding, University of Life Sciences, Poznań, Poland; University of Cincinnati, United States of America

## Abstract

Three *Sus scrofa Piwi* genes (*Piwil1*, *Piwil2* and *Piwil4*) encoding proteins of 861, 985 and 853 aminoacids, respectively, were cloned and sequenced. Alignment of the Piwi proteins showed the high identity between *Sus scrofa* and *Homo sapiens*. Relative transcript abundance of porcine *Piwil1*, *Piwil2* and *Piwil4* genes in testes, ovaries and oocytes derived from sexually immature and mature animals was examined using Real-Time PCR. Expression of the three *Piwi* mRNAs was proved to be tissue specific and restricted exclusively to the gonads. In testes of adult pigs the highest relative transcript abundance was observed for the *Sus scrofa Piwil1* gene. On the other hand, in testes of neonatal pigs the *Piwil1* transcript level was over 2–fold reduced while the level of *Piwil2* transcript was higher. As regards the expression of the *Piwil4* transcript, its level was 34-fold elevated in testes of neonatal piglet when compared to adult male. In ovaries of prepubertal and pubertal female pigs transcript abundance of the three *Piwi* genes was significantly reduced in comparison with testes. However, similarly to testes, in ovaries of neonatal pigs the *Piwil*2 gene was characterized by the highest relative transcript abundance among the three *Piwi* genes analysed. In prepubertal and pubertal oocytes *Piwil1* transcript was the most abundant whereas the expression of *Piwil4* was undetectable. We also demonstrated that expression of piRNA occurs preferentially in the gonads of adult male and female pigs. Moreover, a piRNA subset isolated from ovaries was 2–3 nucleotides longer than the piRNA from testes.

## Introduction

Gametogenesis including spermatogenesis and oogenesis is a very complex process regulated by a large number of genes. It is estimated that over 100 genes are involved in oogenesis and more than 1000 genes in spermatogenesis [1], [2]. It has recently been demonstrated that a family of small RNAs (miRNA, piRNA) play an important role in gene regulation during gametogenesis. miRNAs may control many physiological processes in the cell by modulating transcription, translation, mRNA stability and transport [3]. The recently discovered piRNAs appeared to be expressed in gonads and to originate from transposons and repetitive sequences of the germ cells [4]–[6]. Besides, piRNAs seem to occur only in the form of ribonucleotide-protein (RNP) complexes with Piwi protein partners. This phenomenon has been confirmed in many organisms from fruit fly to mouse with the presence of such RNP complexes detected by immunoprecipitation [7], [8].

RNP complexes formed by small RNA and protein contain mainly the evolutionarily conserved Argonaute proteins. Using bioinformatic analysis, the Argonaute proteins may be classified into two protein subfamilies - Ago and Piwi [9]. Ago proteins are widely distributed among all types of cells and they preferentially bind siRNA and miRNA, whereas the presence of the Piwi proteins is likely to be restricted to the germ cells [4], [10].

Mammalian Argonaute proteins which are approximately 90 kDa in size possess two characteristic domains PAZ (Piwi, Argonaute and Zwille) and PIWI. The PAZ domain consists of 100–200 amino acid residues and preferably binds single stranded RNAs. The PIWI domain is larger (400–600 amino acids) and exhibits structural similarity to the RNAse H domain [9], [11], [12]. Some of the Argonaute proteins equipped with the RNase H domain demonstrate ribonuclease activity. They show nucleolytic specificity towards double-stranded RNAs and cleave RNA duplex in a region formed by small RNAs associated with mRNA [13]. Moreover, some Argonaute proteins contain the MID domain which typically contains about 150 amino acids that are involved in binding of the 5′-phosphate group of small RNAs [11].

The expression of several Ago and 2–4 Piwi proteins has been previously described for several animal species. Fruit fly (*D. melanogaster*) posseses three Piwi proteins, Aubergine, Piwi and Ago2 [14], [15]. Also in rodents three distinct Piwi proteins have been identified [namely *Mus musculus* Piwil1 (Miwi), Piwil2 (Mili), and Piwil4 (Miwi-2); *Rattus norvegicus* Piwil1 (Riwi), Piwil2 (Rili) and Piwil4 (Riwi-2) [16]–[18]. Unlike rodents, frog and zebrafish are characterized by the presence of only two Piwi proteins: *Xenopus leavis* Piwil1(Xiwi), Piwil2 (Xili) and *Danio rerio* Piwil1 (Ziwi), Piwil2 (Zili) [19], [20]. Bioinformatic analysis of the human genome has revealed three Piwi proteins: Piwil1 (Hiwi), Piwil2 (Hili), Piwil4 (Hiwi-2), are similar to those found in other mammals and the Piwil3 protein which has not yet been detected in any organism than human [21].

Currently, only the fruit fly and mouse Piwi proteins involved in spermatogenesis have been well characterized. *Mus musculus Piwil1* and *Piwil2* genes are expressed in the testes of adult males [4], [5]. In contrast to these two *Piwi* genes, *Piwil4* is preferentially expressed in the testes of prenatal and neonatal males. Disturbances in the expression of murine Piwi proteins can have dramatic consequences in the course of spermatogenesis and lead to male infertility [6]. For example, knockout of the mouse *Piwil1* gene leads to arrest in early spermiogenesis, thus confirming an important role of Piwil1 protein in this process [4], [5]. Loss of Piwil2 protein leads to a significant reduction in the piRNA population. The Piwil4 mutation also affects expression of piRNAs. Interestingly, murine Piwil1 and Piwil2 are cytoplasmic proteins whereas Piwil4 operates in the nucleus [22], [23]. Furthermore, mutation of the murine *Piwil2* gene leads to the occurrence of Piwil4 protein in the cytoplasm. On the other hand, in homozygous mice deprived of the *Piwil4* gene, Piwil2 protein was still detectable in the cytoplasm. The observations indicate that the Piwil2 protein may direct the nuclear localization of Piwil4 protein but there is no reverse process. Additionally, Piwil2 and Piwil4 proteins differ in compartmentalization, and are located in distinct types of cytoplasmic granules: Piwil2 in pi-bodies and Piwil4 in piP-bodies [23], [24].

As far as the fruit fly is concerned, three Piwi proteins (Ago3, Piwi, Aub) have been identified in adult spermatic cells [6], [22]. Interestingly, the fruit fly Piwi proteins have been detected not only in germ cells, as has been the case with mouse, but also in some types of somatic cells [12]. It has recently been demonstrated that in contrast to mice, mutation of any *Drosophila* Piwi proteins resulted in female sterility [25].

A growing body of evidence suggests the prominent role of Piwi proteins in germ cell development and their indispensability to the repression of the transposon activity [5]–[7], [14]. This process is well recognized in *Drosophila* as a ping-pong mechanism of transposon silencing. According to this scheme, an unknown nuclease transforms a long single-stranded piRNA precursor transcript into antisense primary piRNAs. Piwi and Aub proteins bind these antisense primary piRNAs which are guided to a transposon target, generating the 5′-end of the sense secondary piRNAs. Then, Ago3 protein binds to secondary piRNAs and generates the 3′-end of the new piRNAs derived from piRNA cluster transcripts. In addition, the nucleolytic activity of the *Drosophila* Piwi proteins has been proven *in vitro* [26]. As Piwi proteins specifically cleave transposon transcripts, they are involved not only in the biogenesis of piRNAs but they are also responsible for silencing transposons activity [14], [27]. Numerous data confirm that the two processes (transposons silencing and piRNA biogenesis) are probably coupled, and therefore the ping-pong cycle seems to generate piRNAs and destroy transposon mRNAs at the same time [14], [27].

However, the majority of the published data on the expression of mammalian Piwi proteins and piRNA are limited to mouse testes. In the presented study, we compared the expression of the three *Piwi* genes and piRNAs in testes and ovaries of neonatal and adult pigs. In addition, we investigated *Piwi* expression in oocytes of prepubertal and pubertal female pigs. Our data clearly show differential expression of *Piwi* and piRNAs in these different tissues during mammalian development.

## Materials and Methods

### Experimental material

In the present study, we used testes and ovaries collected from male and female pigs (*Sus scrofa domestica*) at different age and sexual maturity. Ovaries from neonatal, prepubertal and pubertal females (age of 3 days, 6 and 12 months, respectively) were collected. Before dissecting a pair of ovaries, we additionally evaluated the corresponding uterus in order to distinguish between young and multiparous females. Testes were collected from two males of Line 990: 1) a neonatal male (3-day-old) and 2) a mature male (2 years old). Both males originated from Experimental Station of The National Institute of Animal Production (Pawlowice, Poland) and were slaughtered under supervision of a certified veterinary doctor. Several tissues including testis, kidney, brain, heart, spleen, lung, stomach, intestines, liver and muscles from neonatal male and testis from mature male were isolated immediately after slaughter, frozen in liquid nitrogen and stored at −80°C until analysis. This study was conducted under permission National Commission for the Ethics of Animal Experimentation, Local Committee in Poznań - permission number 70/2008.

### Collection of cumulus-oocyte complexes

Ovaries were transported to the laboratory in a flask within 2–3 hours after recovery. Ovaries were divided into two groups based on their morphology: 1) pubertal and 2) pre-pubertal as described earlier [28]. Pubertal ovaries were characterized by the presence of several bigger follicles 3–6 mm in diameter as well as corpus luteum or corpus albicans on the surface. Pre-pubertal ovaries possessed only small 2–4 mm follicles. Cumulus-oocyte complexes (COCs) were aspirated from 2–5 mm follicles with a syringe, placed in the Hepes-talp medium and morphologically evaluated under a stereomicroscope. Only COCs with proper morphology (at least 3 layers of compact cumulus cells and evenly granulated ooplasm) were included into further procedure.

### RNA isolation and cDNA synthesis

Total RNA from 11 different pig tissues (500 mg of testis, kidney, brain, heart, spleen, lung, stomach, intestines, liver, muscles from piglet and testis from adult) and oocytes (25 oocytes/sample) was extracted using the RNAqueous-Midi Kit (Ambion) and High Pure miRNA Isolation Kit (Roche Diagnostics GmbH, Germany) respectively, according to the manufacturer's instructions. About 2 µg total RNA from tissues was reverse transcribed, using oligo(dT)18 primer and SuperScript III Reverse Transcriptase (Invitrogen). RNA and primer (3.25 µM) were mixed in a 11 µl reaction volume, incubated for 5 minutes at 65° and placed on ice for 2 min. Reverse transcription was carried out with 40 U/µl of SuperScript III reverse-transcriptase, 0.5 mM dNTP mix, 1×buffer (50 mM Tris-HCl pH 8.3, 75 mM KCl, 3 mM MgCl_2_), 5 mM DTT and 20 U/µl of RNase Inhibitor (Promega) in a final reaction volume of 20 µl at 50°C for 1 hour. Finally, the reaction was inactivated at 70°C for 15 min and 2 U of RNase H (Ambion) was added to each sample followed by incubation for 20 min at 37°. The reactions were stored at −20°C until PCR amplification. The total RNA isolated from each pool of oocytes was used for reverse transcription using Transcriptor High Fidelity cDNA Synthesis Kit (Roche). The samples were incubated at 70°C with a primer mix (random hexamers and oligo-dT) for 10 minutes followed by 30 minutes incubation in an RT mix (1× reaction buffer – 50 mM Tris-HCl, 30 mM KCl, 8 mM MgCl_2_, pH 8.5, 1 mM dNTP, 1 U/µl Protector RNase Inhibitor, 5 mM DTT, 0.5 U/µl Reverse Transcriptase). Finally, the enzyme was inactivated by heating at 80°C for 5 min and cDNA was stored at −20°C prior to analysis.

### Cloning of Piwi genes

The *Sus scrofa Piwil1*, *Piwil2* and *Piwil4* coding regions were amplified using proof reading *Pfu* DNA polymerase (Fermentas). The primers used to amplify the three Piwi genes and PCR conditions are shown in Table S1. The PCR product for *Piwil1* was purified using QIAquick PCR Purification Kit (Qiagen). Amplification of *Piwil2* was conducted with 2.5% DMSO followed by purification of the PCR product from agarose gel using phenol extraction. The *Piwil4* coding sequence was obtained in two PCR reactions. In the first one conducted with 1 M betaine, primers hybridized to 5′ and 3′ UTR of *Piwil4* cDNA and a shorter proper product was obtained by reamplification. *Piwil4* product was purified from agarose gel using DNA Gel Extraction Kit (Millipore). The blunt-ended PCR products were ligated into pET-151/D TOPO vectors and were used to transform TOP10 chemically competent *E. coli* cells according to the vendor's instructions (Invitrogen, Carlsbad, CA, USA). Positive colonies were isolated on ampicillin agar plates and grown for plasmid isolation. Plasmid DNA was isolated using EZ-10 Spin Column Plasmid DNA MiniPreps Kit (Bio Basic) according to the manufacturer's instructions. The constructs were sequenced in both directions using an automated DNA sequencer.

### Sequence and comparative analysis of Piwi family

Three porcine coding sequences of *Piwi* genes were sequenced and deposited in the GenBank. Piwi protein sequences from *Sus scrofa* and other mammals such as *Homo sapiens*, *Mus musculus*, *Rattus norvegicus*, *Macaca mulatta* and *Pan troglodytes* were aligned by ClustalW.

### Gene expression analysis by a standard RT-PCR procedure

The reverse transcription PCR was used to analyze the expression patterns of the three Piwi genes in different tissues of pig. The primers for *Piwil1*, *Piwil2*, *Piwil4* and *ACTB* genes and the amplification conditions are shown in Table S2. PCR primers used in this experiment were expected to amplify cDNA of 732, 857, 592 and 483 nucleotides, respectively.

### Relative transcript abundance quantified by real-time PCR analysis

RT PCR experiments were carried out according to the manufacturer's protocol using LightCycler® FastStart DNA Master SYBR Green I (Roche), 0.5 µM primers and 1 µl of each oocyte/testis/ovary cDNA sample. The reaction conditions were as follows: 1 cycle at 95°C for 10 min followed by 45 cycles of 95°C for 15 s, primer specific temperature for 10 s and 72°C for 10 s. Each run was finished with melting curve analysis in order to ensure proper amplification and exclude primer-dimer formation. Altogether, four genes were included into analysis: three genes of interest *Piwil1*, *Piwil2*, *Piwil4* and *β actin* as a reference gene. All information on primers used in this study are listed in Table S3. All samples were tested in duplicate. Transcript quantitation was based on the relative standard curve method. For each investigated gene, a standard curve from tenfold dilutions of corresponding PCR product of known concentrations was built. Samples of standard DNA of known concentration were stored at −20°C. Each real time PCR experiment included one sample with standard DNA which was used for final calculations. The relative transcript abundance of each gene of interest was calculated by dividing the quantity of the target gene by the quantity of the reference gene.

### Small RNA isolation and PAGE analysis

Small RNA samples from testes and ovaries were isolated using mirVana miRNA isolation kit according to the manufacturer's instruction (Ambion). 100–250 mg of tissue was homogenized in 10 volumes of Lysis/Binding buffer. A 1/10 volume of miRNA Homogenate Additive was added and incubated on ice for 10 min. Total RNA was extracted by adding an equal volume of Acid-Phenol:Chloroform. Small RNA was extracted from the total RNA using a filter cartridge with 100 µl of preheated elution solution. The concentration of small RNA was measured using a NanoDrop spectrophotometer. The low-molecular-weight RNA (5–8 µg) was separated on the SYBR-gold-stained 15% denaturing polyacrylamide gel.

### Construction of small RNA cDNA libraries

The low-molecular-weight RNA from testes and ovaries was separated on denaturing polyacrylamide gel. Fractions corresponding to piRNAs from both tissues were recovered from the gel by elution with 0,3 M sodium acetate buffer and cloned using miRCat, Small RNA Cloning Kit according to the manufacture's instruction. The 3′ and 5′ cloning linkers were ligated to purified piRNAs. Then reverse transcription of the linkered piRNAs was carried out followed by PCR amplification. Amplicon pool was digested with *Ban I* restriction endonuclease and concatamerization reaction was carried out using T4 DNA Ligase. Then the concatamer ends were filled with adenosine nucleotides and reaction was cleaned using QIAQuick PCR clean up column (QIAQEN). Cloning was done using TOPO TA Cloning Kit (Invitrogen). Ligation reaction of concatamers and pCR2.1-TOPO vector was transformed into One Shot TOP10 competent bacteria and plated onto LB plates containing ampicillin and X-gal for blue-white color screening. After overnight growth at 37°C pale blue and white colonies were screened by PCR using Taq DNA Polymerase. After 30 PCR cycles products were analyzed on a 1.5% agarose gel. Vectors from clones containing insert of desired length were isolated using EZ-10 Spin Column Plasmid DNA Kit (Bio Basic Inc.) and sequenced.

### Bioinformatic analysis

DNA sequences were analyzed to locate small RNA sequences in the cloning vector. Each small RNA was located in the pig genome using the BLAST program in the UCSC Genome Browser. The strand, as well as its start and end nucleotides in the genome were determined according to the BLAST results. To identify piRNAs, all small RNAs were analyzed using the piRNA Database tool (Table S4 and S5).

## Results

### Identification of the *Piwi* family in porcine genome

We chose the domestic pig (*Sus scrofa*) as the model organism for characterization of Piwi proteins because this organism is biologically more relevant to human than previously studied rodents (mouse, rat) or other model organisms (fruit fly or zebrafish).

To identify members of the *Piwi* genes in the porcine genome, the genomic sequence derived from databases such as NCBI relevance and Ensemble were searched using the BLAST program. We searched for homology between putative porcine Piwi proteins and the previously described sequences of the human and mouse *Piwi* family. Having found homology, we postulated expression of the three porcine *Piwi* genes. Putative sequences of the porcine *Piwi* genes were cloned into a plasmid vector and sequenced using a standard automatic DNA sequencer. Several differences in amino acid sequences between previously deposited Piwi proteins were found (summarized in Table 1). The amino acid sequences of the three porcine Piwi proteins obtained as a result of our studies were deposited in the GenBank (accession No. *Piwil1* – JX036536, *Piwil2* - JX036537 and *Piwil4* - JX036538). It has to be mentioned that on the nucleotide level many more differences were found. However, they are not reflected in the amino acid sequence therefore they may be treated as nucleotide polymorphisms.

**Table 1:** Differences in aminoacid sequences of *Sus scrofa* Piwi proteins.

Protein	Sequence deposited in NCBI and Ensemble data bank gene prediction method	Line 990 pig	Taihu pig Zhou *et al.* [29]
**Piwil1**	**189aa – Glu**		**189aa - Gly**
	**231aa – Asn**		**231aa – Ser**
	**605aa – Ala**		**605aa - Thr**
**Piwil2**	**lack of 11 aminoacids**	**11 additional aminoacids (170–181)**	
	**435aa – Asn**		**435aa - Ser**
**Piwil4**	**69aa – Ala**	**69aa - Thr**	**N/A**
	**191aa - Ala**	**191aa - Val**	

### Evolutionary conservation of the amino acid sequences of mammalian Piwi proteins

The analysis of the open reading frame of the *Sus scrofa Piwi* genes yielded the following sizes of amino acid sequences of the porcine Piwi proteins: Piwil1 – 861 aa, Piwil2 – 985aa and Piwil4 – 853aa, respectively (Table 2). Furthermore, the alignment of the *Sus scrofa* Piwi sequences to Piwi sequences of the selected organisms: Hominidae [human (*Homo sapiens*) rhesus (*Macaca mulatto*) chimpanzee (*Pan troglodytes*)] and Rodents [mouse (*Mus musculus*) rat (*Rattus norvegicus*)] was carried out using the ClustalW program. The highest identity (91-86%) with other mammalian Piwi sequences was observed in the case of the porcine Piwil1 and Piwil2 proteins (Table 2). Lower identity (Hominidae 80–81%, Rodents 74–75%) was observed with regard to the porcine Piwil4 protein.

**Table 2:** Comparison of the identity (%) of amino acid sequences of three *Sus scrofa* Piwi proteins with known Piwi proteins of other mammals.

Protein	Organism	Length (aa)	Organism	Length (aa)	Score (%)
**Piwil1**	*Sus scrofa*	861	*Homo sapiens*	861	96
	*Sus scrofa*	861	*Macaca mulatta*	861	96
	*Sus scrofa*	861	*Mus musculus*	862	98
	*Sus scrofa*	861	*Pan troglodytes*	861	96
	*Sus scrofa*	861	*Rattus norvegicus*	862	98
**Piwil2**	*Sus scrofa*	985	*Homo sapiens*	973	86
	*Sus scrofa*	985	*Macaca mulatta*	973	87
	*Sus scrofa*	985	*Mus musculus*	971	84
	*Sus scrofa*	985	*Pan troglodytes*	973	86
	*Sus scrofa*	985	*Rattus norvegicus*	971	85
**Piwil4**	*Sus scrofa*	853	*Homo sapiens*	852	81
	*Sus scrofa*	853	*Macaca mulatta*	852	80
	*Sus scrofa*	853	*Mus musculus*	878	66
	*Sus scrofa*	853	*Pan troglodytes*	852	80
	*Sus scrofa*	853	*Rattus norvegicus*	848	74

The table was based on ClustalW alignment of the amino acid sequences of Piwi proteins. The GenBank accession numbers of these sequences are: ***Mus musculus*** - NP_067286 (Piwil1), NP_067283 (Piwil2), NP_808573 (Piwil4); ***Homo sapiens*** - NP_004755 (Piwil1 isoform 1), NP_001129193 (Piwil2), NP_689644 (Piwil4); ***Macaca mulatta*** - NP_001182640 (Piwil1), NP_001182592 (Piwil2), NP_001182443 (Piwil4); ***Pan troglodytes*** - XP_001137815 (predicted Piwil1 isoform 1), XP_528083 (predicted Piwil2), XP_001143022 (predicted Piwil4 isoform 2); ***Rattus norvegicus*** - NP_001102323 (Piwil1), NP_001100746 (Piwil2), Q4G033 (Piwil4).

We also determined the chromosomal locations of three *Sus scrofa Piwi* genes: *Piwil1*, and *Piwil2* mapped to the porcine chromosome 14 whereas *Piwil4* mapped to chromosome 9. Location of the *Piwi* genes in the pig is different from other mammals. The human *Piwi* have been mapped to the following chromosomes: *Piwil1* - 12, *Piwil2* - 8 and *Piwil4* – 11. Interestingly, the murine - *Piwil2* and *Piwil4* genes match to the same chromosome as *Sus scrofa Piwi*. Only mouse *Piwil1* is mapped to chromosome 5. The observed variation in chromosomal localizations of *Piwi* genes between pig, humans and mouse could be attributed to the variation in chromosome numbers 46 in humans, 40 in pig and 38 mouse. Moreover, despite a similar chromosomal localization of the porcine and murine *Piwi* genes, the highest identity of amino acid sequences was observed for porcine and human proteins.

**Figure 1:**
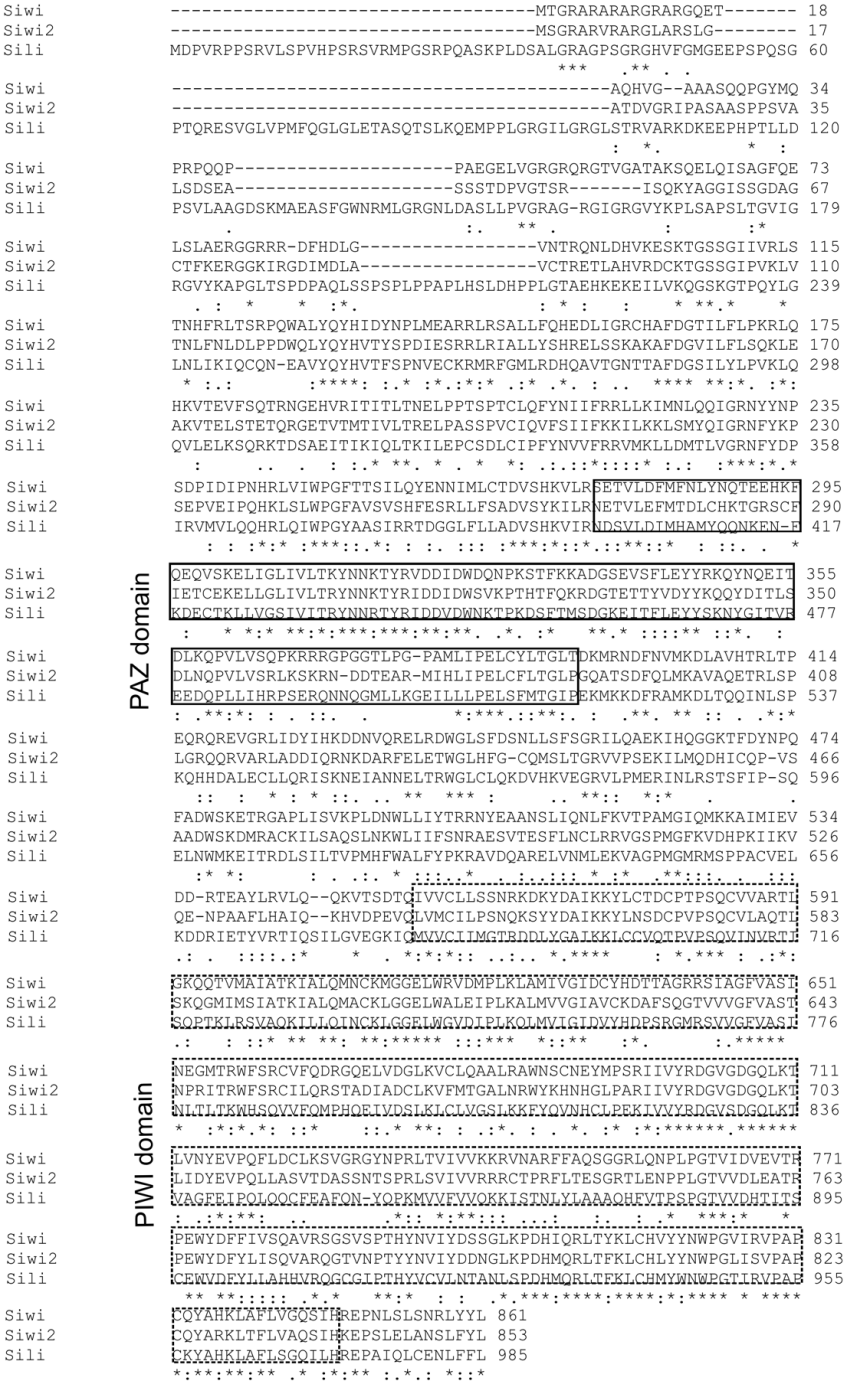
Alignment of the *Sus scrofa* Piwi proteins – Piwil1, Piwil2 and Piwil4.

Comparison of the amino acid sequences of the *Sus scrofa* Piwi proteins revealed two conserved domains - PAZ and PIWI (Fig. 1). The PAZ domain identified in the middle of the Piwi protein sequences also occurs in the Ago proteins and binds the 3′-end of the small RNAs (miRNA, sRNA, piRNA) [9], [11], [12]. Recent structural studies utilizing crystallography and NMR reveal that the PAZ domain of murine Piwi1 and human Piwil2 proteins forms stable complexes with cognate piRNAs [30], [31]. In addition the PIWI domain of some Ago proteins exerts slicer activity and cleaves the RNA chain [32].

### Tissue specific expression of the porcine *Piwi* genes family

**Figure 2:**
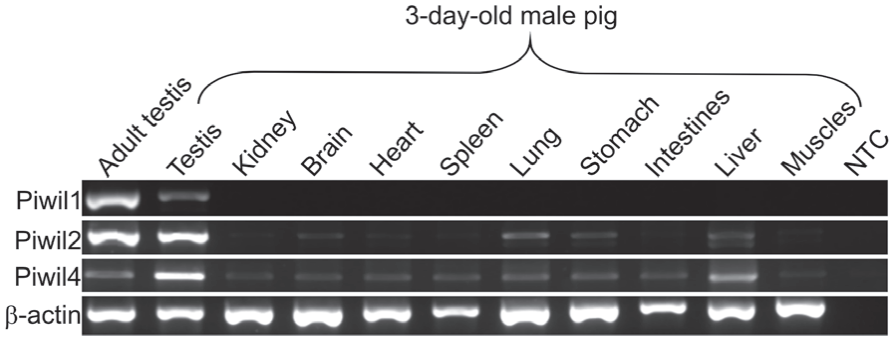
Tissue specific expression of *Piwi* genes in testes of adult and neonatal pigs as well as in nine other somatic tissues of neonatal male. Expression of *Piwil1*, *Piwil2* and *Piwil4* was detected by reverse transcription PCR. Expression of *β-actin* was used as the inner control. NTC – non template control.

Several porcine tissues were analyzed by standard PCR (testis of adult males as well as testis, kidney, brain, heart, spleen, lung, stomach, intestines, liver and muscles of neonatal males). The three porcine *Piwi* genes were expressed specifically in testes (Fig. 2). We also observed a very low relative transcript abundance of the *Piwil1* gene in lung and of *Piwil2* and *Piwil4* in liver. However, the expression of the three *Piwi* genes in tissues other than testis was not detected by more sensitive techniques, such as Real Time PCR (data not shown). These data are consistent with the previously published evidence that expression of the *Piwi* genes in male mice is restricted to testes [8].

### An analysis of the relative transcript abundance of *Piwi* in porcine testes

**Figure 3:**
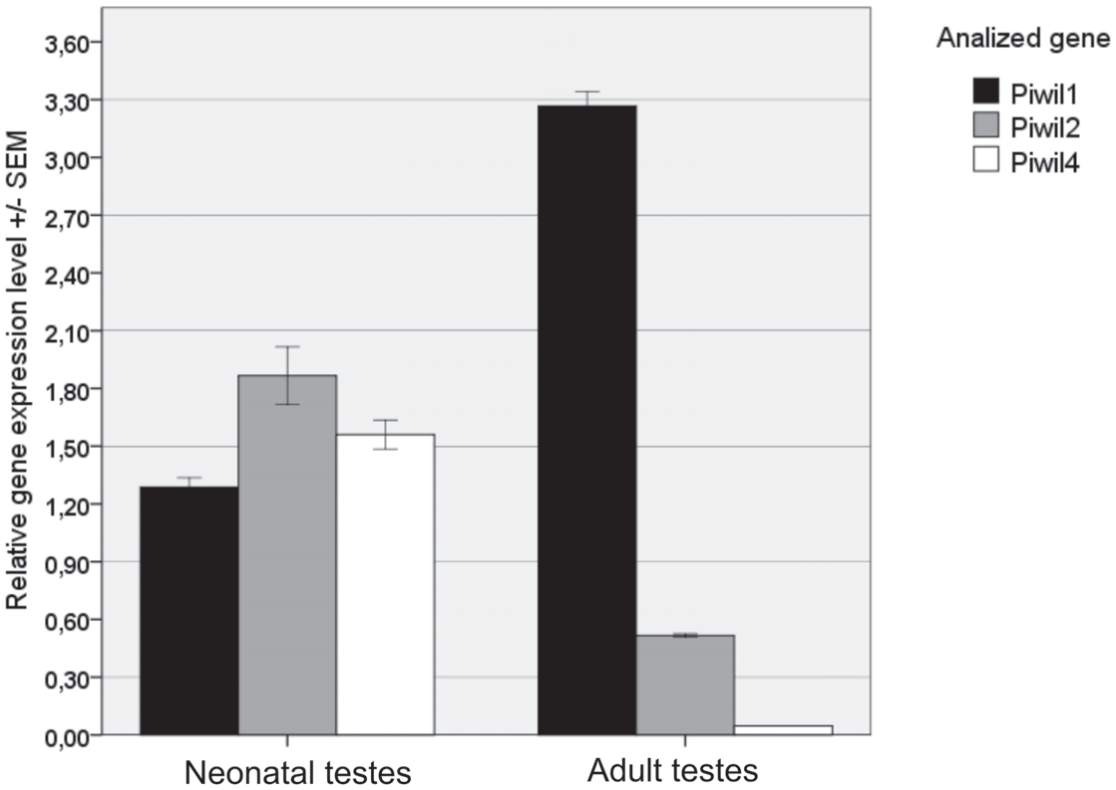
Expression levels of *Sus scrofa Piwi* family genes in testes of neonatal and adult animals. Total RNA extracted from the tissues was examined by real-time PCR. Relative transcript abundance of *Piwil1*, *Piwil2* and *Piwil4* genes was normalized to that of *β-actin* as a reference gene.

Comparison of the relative transcript abundance of the *Piwi* in testes of sexually mature and immature male pigs revealed that *Piwil4* had the greatest difference in expression level (Fig. 3). The *Piwil4* transcript was very abundant in testes of the neonatal animal, but was significantly reduced (almost 34 –fold) in testes of a sexually mature, 2 year old male pig. Protein expression of the *Piwil2* was also reduced (5–fold) in testes of an adult male in relation to a neonatal piglet. Unlike the relative transcript abundance of *Piwil4* and *Piwil2* genes, expression of the *Piwil1* was higher (3-fold) in testes of adult than in neonatal pig.

Abundance of the *Piwi* transcripts in mouse and pig resulted in several differences in the expression profile. Expression of the murine *Piwil4* (*Miwi2*) was shown to be restricted to a narrow window of fetal development, from day 18 post insemination to day 3 *post partum* [33], [34]. In our studies, a low level of the *Piwil4* transcript was detected in testes of adult male. As regards the murine Piwil2 protein, its expression starts in germ line cells and finishes about 20 days *post partum*. Unlike in the mouse, porcine *Piwil2* transcript is still very abundant in testes of adult male as shown in the present study. As far as the murine *Piwil1* (*Miwi*) protein is concerned, its expression is detected several days after birth. We found a high *Piwil1* level in porcine testes one day after birth.

### The comparison of Piwi expression of the *Piwi* genes in porcine ovaries

Oogenesis is a complex process which starts in the fetal gonad with mitotic divisions of the germ cells (e.g. on day 13^th^ of porcine embryo development) and ends up with oocyte ovulation in the ovary of pubertal female [35]. Oocytes enclosed by ovarian follicles stay in a close relationship with follicular cells which support their growth and maturation. Interactions between the oocyte and supporting follicular cells involve several genes encoding, for example, paracrine factors expressed locally in the ovary.

**Figure 4:**
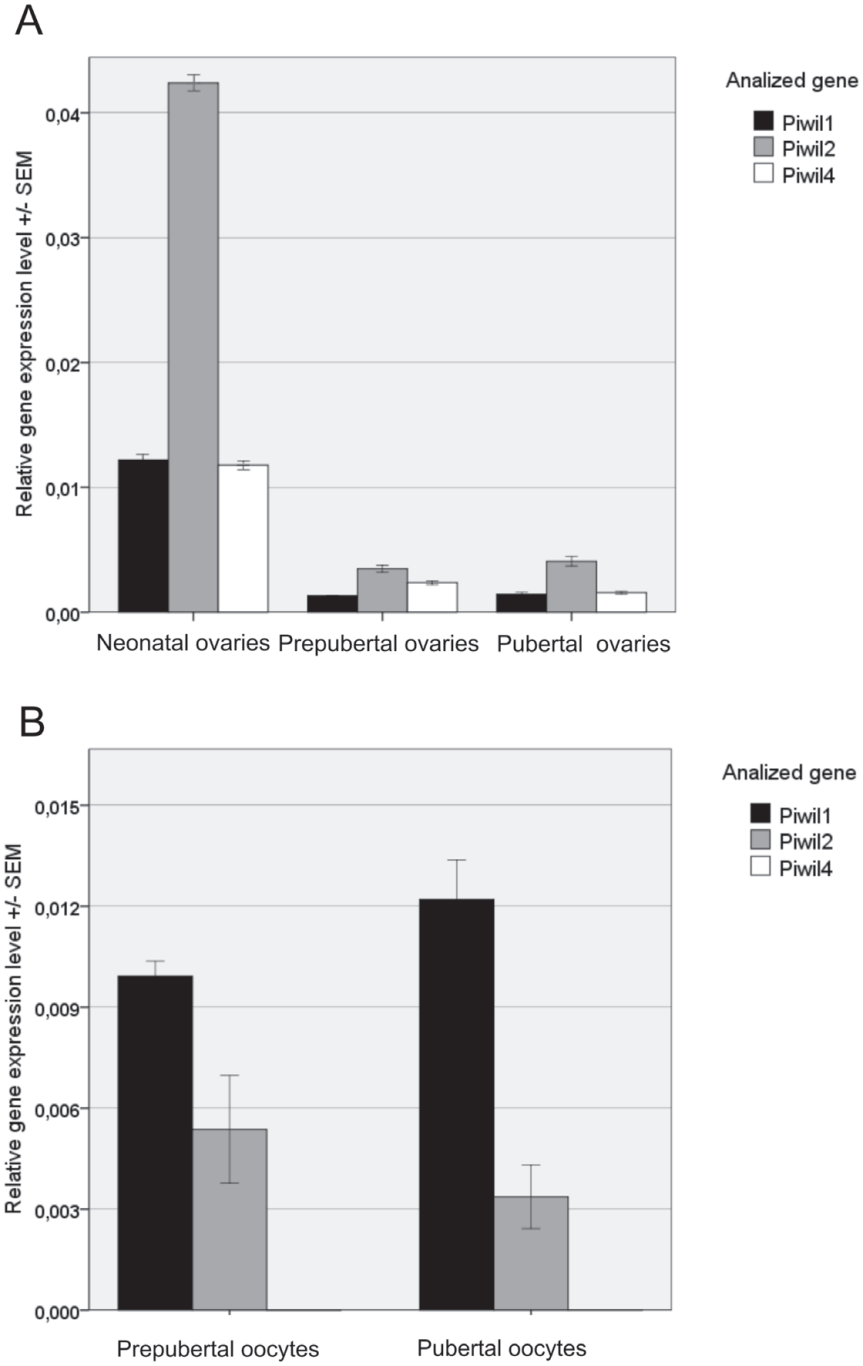
Expression levels of *Sus scrofa Piwi* family genes in ovaries of neonatal, prepubertal and pubertal females (A) and oocytes of prepubertal and pubertal females (B). Total RNA extracted from indicated tissues was examined by real-time PCR. Relative transcript abundance of *Piwil1*, *Piwil2* and *Piwil4* was normalized to that of *β-actin* as a reference gene.

We were interested in whether Piwi proteins and piRNAs associated with them are expressed in porcine ovaries and whether the expression of the *Piwi* genes is altered during *Sus scrofa* development. To this end, ovaries from females differing in age and sexual maturity were investigated (3 day old neonatal females as well as 6 month old prepubertal and 1 year old pubertal females. In general *Piwi* expression was of lower efficiency in porcine ovaries than in testes (Fig. 4A). For example, we found the highest difference in *Piwil1* relative transcript abundance in testes of neonatal male compared to the ovaries of adult female pigs, a difference of about 2000-fold. The lowest difference for the expression of *Piwil2* in testes in relation to ovaries of neonatal pigs was 43-fold. In mice the expression of *Piwi* in testes has also been shown to be more efficient than in ovaries [36]. Besides, expression of *Piwi* genes was generally higher in ovaries of neonatal pig than in ovaries of adult animals. The expression level of *Piwil2* was, at least 10-fold higher in ovaries of neonatal compared to pubertal females Moreover, there was no difference in *Piwi* genes expression in the ovaries of prepubertal and pubertal females.

### An analysis of *Piwi* expression in porcine oocytes

Comparing the expression of the *Piwil1*, *Piwil2* and *Piwil4* genes in oocytes derived from prepubertal and pubertal females, the relative *Piwi* transcript abundance was much lower than in testes and even several times lover than in ovaries (Fig. 4B). Moreover, expression of *Piwi* genes was similar in both prepubertal and pubertal oocytes. In the case of pubertal oocytes, 20% higher expression of the *Piwil1* was detected. On the other hand, the relative transcript abundance of *Piwil2* in both kinds of oocytes was similar. Interestingly, regardless of the sexual maturity of the female, no *Piwil4* transcript was found in oocytes.

### Abundance of piRNA in gonads of sexually immature and mature pigs

An outstanding feature of Piwi proteins is the formation of complexes with piRNAs. Murine germ cells in testes contain a large amount of various piRNA molecules, calculated to be even three orders of magnitude higher than other small RNA - microRNA [4].

**Figure 5:**
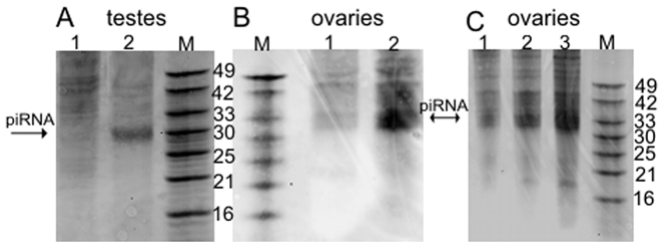
piRNA class of ∼30-nt small RNA identified in *Sus scrofa* testes and ovaries. RNA enriched with low-molecular-weight RNA was isolated from: **A** - testes of neonatal (1) and adult (2) male; **B** – ovaries of neonatal (1) and cyclic (2) females; **C** – ovaries of three-month-old (1), prebupertal (2) and pubertal (3) pigs. Approximately 8 µg of low-molecular-weight RNA was loaded on 15% acrylamide gel and stained using SYBR-gold. M – marker; set of synthetic RNA fragments available in the laboratory.

**Figure 6:**
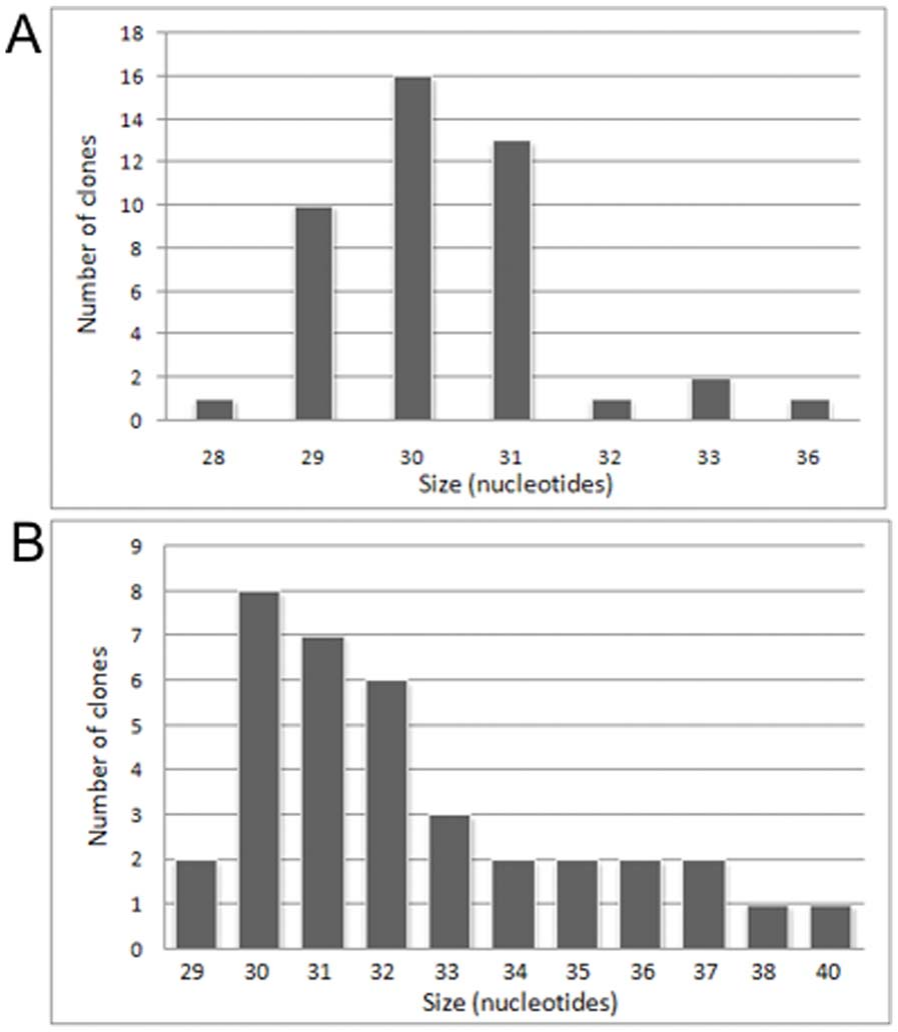
Size distribution of *Sus scrofa* piRNA isolated from testes (**A**) and ovaries (**B**).

In this study, a fraction of small RNAs isolated from porcine neonatal and adult testes and ovaries was analyzed (Fig. 5). In the first step of the analysis, a pool of small RNAs was tested to find out whether they are of piRNA origin. The small RNA pool was cloned and sequenced using standard automatic DNA sequencer. A bioinformatic analysis revealed that the small RNA fraction isolated from porcine gonads is dominated by a 29–31 nucleotides RNA in the testes and 30–37 nucleotides RNA in the case of ovaries (Fig. 6). This agrees with the typical size of mammalian piRNAs [37], [38]. To definitively confirm that the studied small RNA fraction is piRNA, RNA sequences were bioinformatically compared with other piRNA sequences deposited in the piRNA Bank [18]. We found that most of the received sequences of porcine piRNA are identical to the piRNA sequences previously deposited in the piRNA Bank which were isolated from other organisms (fly, fish, mouse, rat and human). (Tables S4 and S5). Interestingly, more than 90% pig ovary specific piRNA and 60% pig testes specific piRNA have sequences identical to the sequences that are deposited in the piRNA Bank. Additionally, all piRNA sequences map to the noncoding part of the porcine genome and murine piRNA shows similar features [37], [38]. In addition, potential contamination of the piRNA pool with fragments of other RNA (mRNA, rRNA, tRNA etc.) was thus excluded. Undoubtedly, the analyzed fraction of small RNAs has therefore confirmed its origin as piRNA.

Upon comparison of the abundance of piRNA in porcine gonads (testes and ovaries) of sexually immature and mature animals some differences were observed (Fig. 5). Major alteration in the expression level of piRNAs in gonads was connected with their abundance in neonatal and mature (older than 1 year) animals. In testes and ovaries of sexually immature pigs piRNAs abundance was low. However, during the development, the expression level of piRNA increased over 40- and 50-fold in testes and ovaries of adult animals, respectively. Besides, we examined the abundance of piRNA in ovaries of prepubertal and pubertal female pigs and no significant difference was noted. Therefore, we suggest that piRNAs have probably a less prominent impact on the development of oocytes in females.

## Discussion

### Nomenclature of Piwi proteins

In the current literature concerning Piwi proteins, historical nomenclature relating to the name of studied organisms has been used. For example, the abbreviations Miwi, Mili, Miwi-2 have been used for mouse Piwi proteins,, whereas Hiwi, Hili, Hiwi-2 and Hiwi-3 have been used for human Piwis. However, using this nomenclature it is difficult to name Piwi proteins derived from other organisms, such as the domestic pig. Annotation of porcine Piwi proteins in a manner similar to murine Piwi as Siwi, Sili and Siwi-2 is impossible because Siwi was previously used to designate Piwi protein from silkworm [39]. In the literature there exists another annotation system, in which genes encoding individual Piwi proteins are numbered 1, 2 etc. while the protein products of these genes are annotated Piwil1, Piwil2 and Piwil4, respectively. We propose the following nomenclature system which combines the Latin systematic name of the studied organism and the Piwi protein number. Hence, the following Piwi proteins are present in mice: *Mus musculus* Piwil1, *Mus musculus* Piwil2, *Mus musculus* Piwil4. Consequently, porcine Piwi proteins are annotated as: *Sus scrofa* Piwil1, *Sus scrofa* Piwil2 and *Sus scrofa* Piwil4. Such an annotation system of Piwi proteins accurately determines the type of Piwi protein studied and helps avoid misunderstandings.

### Developmental expression of Piwi protein

A comprehensive analysis of the expression of the porcine *Piwi* genes in gonads clearly showed that the expression of *Piwil1*, *Piwil2* as well as *Piwil4* depends on the animal developmental stage. All the *Piwi* genes were expressed in testes of neonatal males. However, the expression of *Piwil1* was significantly increased in testes of sexually mature males whereas expression of the two other *Piwi* genes (*Piwil2*, *Piwil4*) was reduced. In the case of both neonatal and adult pig ovaries, the expression of the *Piwi* genes was significantly reduced. However, among ovary *Piwi* transcripts, the abundance of *Piwil2* in neonatal female was elevated.

It is well established that during *Drosophila* spermatogesis Piwi proteins actively participate in transposon silencing and piRNA biogenesis and both these processes are coupled [27]. Recently, a similar mechanism of piRNA biogenesis connected with the function of transposon deactivation has been proposed in mice [40], [41]. Both teams have shown convincing experimental evidence of the involvement of Piwi proteins, i.e. Piwil1 (Miwi), Piwil2 (Mili) and Piwil4 (Miwi2) in the silencing of transposons as well as in the production of piRNA. Replacing the *Mus musculus* Piwil2 and Piwil4 catalytic triad DDH (Asp-Asp-His) with DAH (Asp-Ala-His) motif generates the Piwil2^DAH^ and Piwil4^DAH^ mutant mice in which biogenesis of piRNA is affected during spermatogenesis. In the case of a homozygous Piwil2^DAH^ mouse, piRNAs production has been low and the number of piRNA has been significantly reduced. However, no abnormal piRNA biogenesis pathway in a Piwil4^DAH^ mutant has been observed. Interestingly, homozygous Piwil2^DAH^ mice have been sterile whereas Piwil4^DAH^ mutation has had no impact on female sterility [41] Additionally, Piwil1^ADH^ mutant has been deprived of the slicer activity either *in vivo* or *in vitro*.

**Figure 7:**
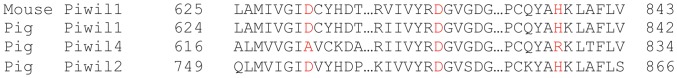
Sequence alignment showing the catalytic triad (DDH motif) of mouse Piwil1 and porcine Piwi family proteins. Catalytic aminoacids are shown in blue.

In testes of neonatal pigs, three Piwi proteins are expressed and presumably they are involved in the process of spermatogenesis, whereas in a sexually adult male a high expression level of only *Piwil1* was detected. A bioinformatic analysis of the amino acid sequence of porcine Piwi proteins revealed the presence of a catalytic slicer motif DDH in Piwil1 and Piwil2 proteins (Fig. 7). Instead of the catalytic domain, Piwil4 contains an ADR (Ala-Asp-Arg) amino acid triad. It should be noted that in homozygous murine mutants Piwil1^ADH^ and Piwil2^DAH^ had no slicer activity towards transposon transcripts and the formation of new piRNAs has been abolished [41]. We therefore suggest that porcine Piwil1 and Piwil2 proteins containing the DDH motif may also exhibit the slicer activity and participate in piRNA biogenesis. Deep sequencing of the *Sus scrofa* piRNA library derived from testes of adult male has shown that most of piRNA sequences are distributed mainly among repeated, intronic and intergenic sequences of the genome, thus confirming their transposon origin [42].

However, *Sus scrofa* Piwil4 protein containing the ADR motif is presumably deprived of the slicer activity. *M.musculus Piwil4* gene is expressed in the embryonic period of life and stops 3 days after birth [23]. In our study, we also noted high expression of *Piwil4* in testes of neonatal male and lower in testes of adult male pig. Knock out of the mouse *Piwil4* gene results in increased expression of transposons due to demethylation in piRNA clusters during the fetal period of life [16], [43], [44]. This means that the piRNA-Piwil4 complexes may regulate the methylation status of transpozons and affect their silencing. This fact points to the differential involvement of Piwil4 protein in gametogenesis in comparison with Piwil1 and Piwil2 proteins. However, the detailed mechanism of involvement of porcine Piwil4 protein in this process needs further study.

### Do Piwi proteins regulate the development of porcine ovaries and oocytes?

The participation of Piwi proteins and their piRNA partners in the development of mammalian oocytes and ovaries is still unknown. Therefore, we analyzed the expression of *Piwi* genes as well as of piRNA in oocytes and ovaries derived from females at different age. As we have shown, the expression of the three *Piwi* genes in neonatal ovaries was at least 40 – fold lower than in neonatal testes. Taking into account these observations we suggest that the role of Piwi proteins in piRNA biogenesis during oogenesis may be less pronounced than during spermatogenesis. In addition, abundance of piRNAs in ovaries of adult female was significantly higher than in neonatal state. Due to the low expression of Piwi proteins, other factors may possibly be involved in the biogenesis of piRNAs in ovaries of adult female. In fact, recently, several other proteins which form complexes with Piwi proteins have been identified. It has been shown that Piwi proteins interact with Tudor domain - containing proteins [45]. In the mouse, 28 Tudor family proteins have been identified and so far the interaction of Piwil1, Piwil2 and Piwil4 with Tdrd1, Tdrd2, Tdrd4, Tdrd6, Tdrd7, Tdrd8, Tdrd9 has been detected [46]. However, the developmental expression profile of Tudor proteins is unknown.

The expression profile of porcine piRNA and Piwi proteins in prepubertal and pubertal oocytes shows similar low abundance for both species. Therefore, the involvement of piRNA – Piwi complexes in oocyte development is unclear. Interestingly, suppression of the other small RNA –miRNA during mouse oocyte development has been reported. [47], [48]. Hence, it is possible that small RNAs (miRNA, piRNA) play a less important role in the oocyte growth. Moreover, a low content of Piwi protein in mouse oocytes and blastocystes has also been mentioned [36]. Moreover, Piwil2^DAH^ and Piwil4^DAH^ as well as Piwil1^ADH^ homozygous mouse male have been sterile whereas these female mutants have remained fertile, which may indicate a limited influence of Piwi proteins on the process of mammalian oogenesis [41].

We showed that *Sus scrofa* piRNA abundance is lower in oocytes than in ovaries. Therefore, it is possible that some piRNAs detected in the ovaries were obtained from somatic cells that accompany the growing oocytes (e.g. follicular cells, ovarian tissue). Supporting evidence comes from several animal species (*M.musculus*, *X.leavis*, *D.melanogaster*) where piRNA origin from the 5′UTR region of specific genes has been observed in somatic cells of the ovaries and testes [49]. Recently, the application of the deep sequencing has revealed a new class of piRNAs in hipocampal neurons [50].

In conclusion, we found nonlinear expression of Sus scrofa *Piwi* genes and piRNA in gonads. In neonatal male, high *Piwil4* transcript abundance is accompanied by low level of piRNA expression. On the other hand, a low abundance of *Piwil1* transcript is accompanied by high piRNA expression in adult ovaries. So presumably, Piwi proteins and piRNA act in a mammalian organism not only as complexes but they may also act separately.

## Supporting Information

Table S1 Set of primers applied for cloning of *Sus Scrofa* Piwi proteins. On the right, PCR conditions optimized to obtain full length of each *Piwi* gene are shown.
(PDF)

Table S2 Primer sequences and PCR reaction conditions used for the analysis of tissue specificity of *Piwi* expression.
(PDF)

Table S3 Primer sequences used in real time PCR experiment.
(PDF)

Table S4 piRNA identified from the testes.
(PDF)

Table S5 piRNA identified from the ovaries.
(PDF)
