# Supplementary material for: Altered Expression of Porcine Piwi Genes and piRNA during Development
Source: PLoS One. 2012 Aug 30;7(8):e43816. doi: 10.1371/journal.pone.0043816 (PMC3431407; doi:10.1371/journal.pone.0043816)
Supplement: Table S1 — Set of primers applied for cloning of Sus Scrofa Piwi proteins. On the right, PCR conditions optimized to obtain full length of each Piwi gene are shown. (PDF) [file pone.0043816.s001.pdf]

Table S1

Gene	Primer sequences	PCR conditions
Piwil1	CACCATGACTGGGAGAGCCAGAG<br>TTAGAGGTAGTAAAGGCGATTTGAC	30 cycles: 95°, 30 s; 65°, 30 s; 72°, 6 min
Piwil2	CACCATGGATCCTGTTCGACCAC<br>TCACAGAAAGAACAGGTTCTCACAC	30 cycles: 95°, 30 s; 62°, 30 s; 72°, 6 min
Piwil4	AAGACACTGTGGGCTCACTGGGAAC<br>CTAAGGATGTCAGTGCTTCGTGTCA	30 cycles: 95°, 30 s; 55°, 30 s; 72°, 5 min
	CACCATGAGTGGACGGGCCCGCGTG<br>TCACAGGTAGAAGAGAGAATTGGCTAATTCC	33 cycles: 95°, 30 s; 68°, 30 s; 72°, 5 min
