# Supplementary material for: Altered Expression of Porcine Piwi Genes and piRNA during Development
Source: PLoS One. 2012 Aug 30;7(8):e43816. doi: 10.1371/journal.pone.0043816 (PMC3431407; doi:10.1371/journal.pone.0043816)
Supplement: Table S2 — Primer sequences and PCR reaction conditions used for the analysis of tissue specificity of Piwi expression. (PDF) [file pone.0043816.s002.pdf]

Table S2

Genes	Primer sequences	PCR conditions
PIWIL1	5’ CTACATCACCAACTTGCTTG 3’<br>5’ AATCGAAGCTCAAACCCCAG 3’	30 cycles: 95° 30 s; 60° 30 s; 72° 1 min
PIWIL2	5’ GTGAAGCAAGGATCAAAAGG 3’<br>5’ ATGAAGGAAAGCTCAGGCAG 3’	30 cycles: 95° 30 s; 60° 30 s; 72° 1 min
PIWIL4	5’ GAAGCATCTTCTAGCACTGACC 3’<br>5’ GAATTTCCACTGGCTCAGAAGG 3’	30 cycles: 95° 30 s; 60° 30 s; 72° 1 min
ACTB	5’ AGAGCAAGAGAGGCATCCTG 3’<br>5’ CGACGTAGCACAGCTTCTCC 3’	30 cycles: 95° 30 s; 60° 30 s; 72° 1 min
