# Supplementary material for: Altered Expression of Porcine Piwi Genes and piRNA during Development
Source: PLoS One. 2012 Aug 30;7(8):e43816. doi: 10.1371/journal.pone.0043816 (PMC3431407; doi:10.1371/journal.pone.0043816)
Supplement: Table S3 — Primer sequences used in real time PCR experiment. (PDF) [file pone.0043816.s003.pdf]

Table S3.

Gene	Primer sequence		Product length [bp]	Annealing temperatu re
Piwil1	Forward<br>Reverse	AAGAAAGCAGATGGCTCTGA<br>CGCATTTTATCAGTTAGACCT	194	60°C
Piwil2	Forward<br>Reverse	CATCATGTACGGCAGGGGTGTG<br>GATGCAAGATTTGTCCTGACAG	199	60°C
Piwil4	Forward<br>Reverse	GTCGTGGATCTGGAAGCAACTC<br>TACAGGTGGCACAGTTTGAAGG	144	60°C
ACTB	Forward<br>Reverse	CAAAGCCAACCGTGAGAAGA<br>GTACCCCTCGTAGATGGGCA	172	60°C
