# Supplementary material for: Altered Expression of Porcine Piwi Genes and piRNA during Development
Source: PLoS One. 2012 Aug 30;7(8):e43816. doi: 10.1371/journal.pone.0043816 (PMC3431407; doi:10.1371/journal.pone.0043816)
Supplement: Table S4 — piRNA identified from the testes. (PDF) [file pone.0043816.s004.pdf]

Table S4

piRNA	Sequence	Length (nt)	Start	End	Hit
piR1	TGGCGTCCGAACAGGGACCTGAACCCTGGACCCTCAGATT	40	24228794<br>9814434<br>72592496<br>50559202<br>24079472<br>22328188<br>9818575<br>9939665	24228827<br>9814467<br>72592529<br>50559235<br>24079505<br>22328221<br>9818608<br>9939698	Human
piR2	GTTCCCATACCGGGAGTCGAACCCGGGCCGCCTGGGTG	38	24760398<br>29171439<br>21192835<br>10249768<br>148851502	24760435<br>29171476<br>21192872<br>10249768<br>148851538	Zebrafish
piR3	CCAGGCGGCCCGGGTTCGACTCCCGGTATGGGAACCA	37	29171439<br>24760401<br>10249768<br>21192838<br>148851505	29171473<br>24760435<br>10249802<br>21192872<br>148851538	Zebrafish
piR4	TTCCCACACCGGGAGTCGAACCCGGGCCGCCTGGGTG	37	21192835<br>148851502<br>10249769<br>24760398<br>29171440	21192871<br>148851538<br>10249805<br>24760434<br>29171476	Zebrafish
piR5	TGCATTGGCCGGGAACCGAACCCGGGCCTCCCGCGT	36	50565564<br>22918478<br>22026330<br>9982529	50565599<br>22918513<br>22026365<br>9982564	Zebrafish

			9983187<br>10072406<br>10073061<br>59476045	9983222<br>10072441<br>10073096<br>59476080	
piR6	AACCCGGGACCTCTCGCACCCGAAGCGAGAATCATA	36	86620909<br>35039929	86620944<br>35039964	Drosophila
piR7	GCTCCCCGTCGGGGAATTGAACCCCGGTCTCCCGC	35	80875449<br>60233310	80875482<br>60233343	Zebrafish<br>Drosophila
piR8	CAGGGGAAAGCGCGAACGCAGTCCCCCACTACCAC	35	60869678<br>103384421<br>33058405<br>33059505<br>33062738<br>33069923<br>33090970<br>69173418<br>69174444<br>32871137<br>32888128<br>32889167<br>32894222<br>32895260<br>32962273	60869712<br>103384455<br>33058439<br>33059539<br>33062772<br>33069957<br>33091004<br>69173452<br>69174478<br>32871171<br>32888162<br>32889201<br>32894256<br>32895294<br>32962307	Lack of similarity
piR9	GTTCCCACACCGGGAGTCGAACCCGGGCCGCCTG	34	21192839<br>10249768<br>48851506	21192872<br>10249801<br>148851538	Zebrafish
piR10	TCCTCGTTAGTATAGTGGTGAGTATCCCCGCCT	33	22776509<br>82399251<br>22687100<br>22722607<br>28246046<br>28249755	22776541<br>82399283<br>22687132<br>22722639<br>28246078<br>28249787	Rat,<br>Drosophila,<br>Human,<br>Zebrafish
piR11	AAGTTCTGATGACCCACTACCATCGGACCAGCC	33	101909568	101909600	Mouse

piR12	AGGCTGATGCCTTATCCATTAGGCAATTGGGTC	33	36016079	36016105	Rat
piR13	GCATTGGTGGTTCAGTGGTAGAATTCTCGCCT	32	22918517<br>22026369<br>9982568<br>9983226<br>10072445<br>10073100<br>103342160<br>59476084<br>103343681<br>99434291<br>50565529<br>18721267	22918548<br>22026400<br>9982599<br>9983257<br>10072476<br>10073131<br>103342191<br>59476115<br>103343712<br>99434322<br>50565560<br>18721298	Zebrafish
piR14	GCATTGGTGGTTCAGTGGTAGAATTCTCGCC	31			
piR15	GCATTGGTGGTTCAGTGGTAGAATTCTCGC	30			
piR16	GTTTCCGTAGTGTAGTGGTTATCACGTTCGCC	32	50565528<br>22918518	50565559<br>22918549	Mouse,<br>Human
piR17	GGCCCAGCACGCTTCCGCTGCGCAACTCTGCT	32	22016218<br>100141173<br>25748935<br>22025582<br>22039701<br>22584933<br>22782671<br>22918074<br>100241400	22016249<br>100141204<br>25748966<br>22025613<br>22039732<br>22584964<br>22782702<br>22918105<br>100241431	Rat,<br>Drosophila,<br>Human
piR18	GCATTGGTCGTTCAGTGGTAGAATTCTCGCCT	32	22918517<br>22026369<br>9982568<br>9983226<br>10072445<br>10073100	22918548<br>22026400<br>9982599<br>9983257<br>10072476<br>10073131	Zebrafish

			103342160<br>59476084<br>103343681<br>99434291<br>50565529<br>18721267	103342191<br>59476115<br>103343712<br>99434322<br>50565560<br>18721298	
piR19	GGCGAACGTGATAACCACTACACTACGGAAAC	32	22315565<br>22561147<br>22848251<br>103321431<br>103324539<br>103360758<br>103109886<br>45218877<br>22825189<br>103306974<br>103337022<br>103357454<br>103373351<br>103376540	22315596<br>22561178<br>22848282<br>103321462<br>103324570<br>103360789<br>103109917<br>45218908<br>22825220<br>103307005<br>103337053<br>103357485<br>103373382<br>103376571	Rat,<br>Human,<br>Zebrafish
piR20	GGCGAGAATTCTACCACTGAACCACCAATGCA	32	50565528<br>22918518	50565559<br>22918549	Zebrafish
piR21	GGCGAGAATTCTACCACTGAACCACCAATGC	31	103343681<br>99434291<br>50565529<br>18721267<br>22918518<br>22026370<br>9982569<br>9983227<br>10072446<br>10073101<br>103342161	103343711<br>99434321<br>50565559<br>18721297<br>22918548<br>22026400<br>9982599<br>9983257<br>10072476<br>10073131<br>103342191	Zebrafish

			59476085	59476115	
piR22	GCGAGAATTCTACCACTGAACCACCAATGC	30	103343681<br>99434291<br>50565529<br>18721267<br>22918519<br>22026371<br>9982570<br>9983228<br>10072447<br>10073102<br>103342162<br>59476086	103343710<br>99434320<br>50565558<br>18721296<br>22918548<br>22026400<br>9982599<br>9983257<br>10072476<br>10073131<br>103342191<br>59476115	Zebrafish
piR23	CGAGAATTCTACCACTGAACCACCAATGC	29	103343681<br>99434291<br>50565529<br>18721267<br>22918520<br>22026372<br>9982571<br>9983229<br>10072448<br>10073103<br>103342163<br>59476087	103343709<br>99434319<br>50565557<br>18721295<br>22918548<br>22026400<br>9982599<br>9983257<br>10072476<br>10073131<br>103342191<br>59476115	Zebrafish
piR24	GCATTGGTGGTTCAATGGTAGAATTCTCGCC	31	103302223	103302253	Zebrafish
piR25	GTCAGGATGGCCGAGCGGTCTAAGGGCTGCG	31	20170687<br>22216041<br>20170279<br>36704541<br>173932953	20170718<br>22216072<br>20170310<br>36704572<br>173932984	Zebrafish
piR26	GCGCCGAATCCTAACCACTAGACCACAGGGA	31	24237454<br>38786758	24237485<br>38786789	Rat,<br>Human,

			103403682<br>65592897	103403713<br>65592928	Zebrafish
piR27	TTAACAATAACCCACAGCCTTCGGACCAACT	31	101923633	101923663	Mouse
piR28	GCGAGAATTCTACCACTGAACCACCCATGCA	31	132073701<br>277622457	132073731<br>277622487	Rat,<br>Zebrafish
piR29	GCGAGAATTCTACCACTGAACCACCCATGC	30			
piR30	GCATTGGTGGTTCAGTGGTAGAATTTTCGC	30			Zebrafish
piR31	GCATGGGTGGTTCAGTGGTAGAATTCTCGC	30	132073702<br>277622458	132073731<br>277622487	Rat,<br>Zebrafish
piR32	TACATTAGCATTTGCTGCTTCCTCCTTGCA	30	40625273	40625302	Rat
piR33	ACGAGAATTCTACCACTGAACCACCCATGC	30			
piR34	GCGAGAATTCTACCACTGAACCACAAATGC	30			
piR35	GCATTGTGGTTCAGTGGTAGAATTCTCGC	29	22918519<br>22026371<br>9982570<br>9983228<br>59476086<br>50565529	22918548<br>22026400<br>9982599<br>9983257<br>59476115<br>50565558	Human
pi36	TTGGCTAAGATCAAGTGTAGTATCTGTTCTT	31	79311923<br>120150384<br>53337925<br>70045065<br>119180842<br>67380052<br>63663234<br>12881812<br>13749154<br>26804963<br>36025806	79311953<br>120150845<br>53337955<br>70045095<br>119180872<br>67380082<br>63663264<br>12881842<br>13749184<br>26804993<br>36025836	Mouse
