# Supplementary material for: Altered Expression of Porcine Piwi Genes and piRNA during Development
Source: PLoS One. 2012 Aug 30;7(8):e43816. doi: 10.1371/journal.pone.0043816 (PMC3431407; doi:10.1371/journal.pone.0043816)
Supplement: Table S5 — piRNA identified from the ovaries. (PDF) [file pone.0043816.s005.pdf]

Table S5

piRNA	Sequence	Length (nt)	Start	End	Hit
piR1	ATGCCCAAGGCTTCCATCTTCTATCTAGAAA	31	7424377	7424406	Human
piR2	TGATCTCGTGCACAGATACTGTTGGTTCCA	30	42960799	42960828	-
piR3	CAGGAATCCTAACCGCTAGACCATGTGGGA	30	21192800<br>148851467<br>10249811<br>98879286<br>24760363<br>29171482	21192829<br>148851496<br>10249840<br>98879315<br>24760392<br>29171511	Zebrafish
piR4	TGCCAAGCCTGGGAACTTCCATATGGCGCA	30	263196213	263196241	-
piR8	TGAACCCTTGGCCTCCTGGCTTGCCCTGTCCC	32	6968806	6968837	-
piR10	TAGCTAAGAGCAAGTAAATTAGCCTGAGAGT	31	7508625	7508655	-
piR12	GGCCATTCATATATCTTCTTTTGTGAAGTGT	31	95574195<br>38439769<br>229842641<br>22278590<br>8970594	95574225<br>38439799<br>229842671<br>22278620<br>8970620	Human<br>Mouse<br>Rat
piR13	ACACAAAGGTAATTTTGTGTATTTACCAACA	31	15120696	15120726	Rat
piR14	GACACTCCAAGTTCGGAGACCTGGACAATTA	31	15105010	15105040	-
piR15	ACACACAATACATTAACATCATTTCTCTTCA	31	117966082	117966111	-
piR16	TAACAGAATGCAGTCTGGTTTCATTTGAGC	30	34501596	34501625	Rat<br>Mouse<br>Human
PiR17	TGGCGTCAAGGACTGTGTTTTAACCCATTA	30	7507722	7507751	-
PiR18	TAATACAGCCCTTGTCCTGCCTCCTGCGG	29	6967095	6967123	-
PiR19	TGGTGACTTGAGCCTTAGTTTCCCTGCAC	29	34484853	34484881	Zebrafish
PiR20	AACACTCATCATCAAAGTCGGGCTTATTG	29	93568325	93568353	-

piR21	TAAACCGCCAGCCAAGATGGGATTTTGCTTT	31	7508683	7508713	-
piR22	TGAATTCATAGTCTTTCTCTCTCTGGTGACC	31	34470745	34470775	-
piR23	GCCGCCAGTTTACACCACAGCAATGTGGGAA	31	54061408	54061437	-
PiR24	TATTTATGTGATTAAAGTGCAAAGCAGTGTC	31	94285852	94285882	-
PiR25	TGCTCTGAACCTCCTCATGTTGCTTACCTGT	31	42961244	42961274	-
PiR26	TGAGTCCTTTGTCTTGCAAAATGCTCGTGG	30	34505404	34505433	-
PiR27	TGAAGGAGCCTGTGAATTGCTCGTGGTGGT	30	117959055	117959083	-
PiR28	TTGCCAGTGATTGGTTTAGGCATGGCCATG	30	6959461	6959490	Human
PiR29	CAGGAATCCTAACCGCTAGACCATATGGGA	30	24760363<br>29171482<br>21192800<br>98879286<br>10249811	24760392<br>29171511<br>21192829<br>98879315<br>10249840	Zebrafish
piR30	ACCTATTACCCATCTTGGACAGGTCCAAACA	31	117968527	117968557	Human
piR31	TACCGTCCATCATGACTGGGAAATGGGGTA	30	95915826	95915855	-
PiR32	TGCTCTCACAGTGATCACCTTGTCCAGAGT	30	42963746<br>42978343<br>43091557	42963775<br>42978372<br>43091586	-
PiR33	TCCCACATGGTCTAGCGGTTAGGATTCCTG	30	98879286<br>21192800<br>10249811<br>148851467<br>29171482<br>24760363	98879315<br>21192829<br>10249840<br>148851496<br>29171511<br>24760392	Zebrafish
PiR34	TTCTAACAAGCTCCCTGGAGAATTCACCCA	30	31978195<br>31880201	31978224<br>31880230	Mouse
PiR35	TGGCTTCTGAGATTGCGTCCAAGAGATGGT	30	34497749	34497778	-
PiR36	TAAGTCAGATGTGGCATTTGCGCAGGTGT	29	34505124	34505152	Human
PiR37	TGAGAACTTAGGTGTTTCCAGTTGGGCCA	29	7433664	7433664	-
PiR38	TACAAAGCATGGTGACCTGTGTGTGTGGA	29	34463718	34463746	Mouse
PiR39	GTGCAGGGAAACTAAGGCTCAAGTCACCA	29	34484853	34484881	Zebrafish

PiR40	TCCACACACACAGGTCACCATGCTTTGTA	29	34463718	34463746	Mouse
PiR41	TCACAACTGAGGTAGTTGGGGAACCAGGT	29	34505603	34505631	-
PiR42	GCCGGAAAGAGCTGCGGTCACACTTTCA	28	50840527	50840554	Zebrafish
PiR43	CAGGCGGCCCGGGTTCGACTCCCGGTATGGGAACCA	36	29171439<br>24760402<br>10249768<br>21192839<br>148851506	29171472<br>24760435<br>10249801<br>21192872<br>148851538	Zebrafish
piR67	TGGACTGGGAATCAGACTTGGAGCGCAATCC	31	59256537	59256567	Rat
PiR44	TCCCATATGGTCTAGCGGTTAGGATTCCTG	30	29171482<br>24760363<br>98879286<br>10249811<br>21192800	29171511<br>24760392<br>98879315<br>10249840<br>21192829	Zebrafish
PiR45	TTCCTTATTAGGAGAACCTTTCCCCTGGTC	30	42961281	42961310	-
PiR46	TCACACCTGCTGCCTGAGGATGGTCCGTC	29	25518925	25518953	Zebrafish<br>Rat
PiR47	GGGGGGGTGTCCCTCGCGGGGGCGCGCCGGGTT	33	251767	251799	-
PiR48	CTCCACCCGCCCTCCGTCCGCGCCCCCTCGCCG	33	252161	252193	-
